# LY294002 Inhibits Intermediate Conductance Calcium-Activated Potassium (KCa3.1) Current in Human Glioblastoma Cells

**DOI:** 10.3389/fphys.2021.790922

**Published:** 2022-01-07

**Authors:** Concetta Caglioti, Federico Palazzetti, Lorenzo Monarca, Raffaele Lobello, Maria Rachele Ceccarini, Rossana Giulietta Iannitti, Roberta Russo, Francesco Ragonese, Chiara Pennetta, Antonella De Luca, Michela Codini, Bernard Fioretti

**Affiliations:** ^1^Department of Chemistry, Biology and Biotechnologies, University of Perugia, Perugia, Italy; ^2^Department of Medicine, Perugia Medical School, University of Perugia, Perugia, Italy; ^3^Relab S.r.l., Genova, Italy; ^4^Department of Pharmaceutical Sciences, University of Perugia, Perugia, Italy; ^5^S&R Farmaceutici S.p.A Bastia Umbra, Perugia, Italy

**Keywords:** LY294002 (PI3K inhibitor), KCa3.1 (intermediate-conductance Ca2+-activated K+ channel), glioblastoma, histamine (H1) receptor, calcium homeostasis, chemio and radioresistance

## Abstract

Glioblastomas (GBs) are among the most common tumors with high malignancy and invasiveness of the central nervous system. Several alterations in protein kinase and ion channel activity are involved to maintain the malignancy. Among them, phosphatidylinositol 3-kinase (PI3K) activity and intermediate conductance calcium-activated potassium (KCa3.1) current are involved in several aspects of GB biology. By using the electrophysiological approach and noise analysis, we observed that KCa3.1 channel activity is LY294002-sensitive and Wortmannin-resistant in accordance with the involvement of PI3K class IIβ (PI3KC2β). This modulation was observed also during the endogenous activation of KCa3.1 current with histamine. The principal action of PI3KC2β regulation was the reduction of open probability in intracellular free calcium saturating concentration. An explanation based on the “three-gate” model of the KCa3.1 channel by PI3KC2β was proposed. Based on the roles of KCa3.1 and PI3KC2β in GB biology, a therapeutic implication was suggested to prevent chemo- and radioresistance mechanisms.

## Introduction

Glioblastomas (GBs) are among the most common tumors with high malignancy and invasiveness of the central nervous system (Holland, 2001; Dionigi et al., 2020; Muir et al., 2020). Typical cell signaling alterations due to gene mutation and chromosomal abnormalities were found in GB. The amplification or activating mutations of the EGFR activity, the overexpression of FGF, FGFR, and PDGF, and/or PDGFR are frequently found in these tumors, and, in general, all of them converge into a constitutive activation of transduction pathways such as phosphatidylinositol 3-kinase (PI3K)/AKT, RAS/MAP kinase, C-MYC, PKC, and STAT pathways (Parsons et al., 2008). PI3K converts phosphatidylinositol 2 phosphates [PI (4,5) (PIP2)] to phosphatidylinositol 3 phosphates [PI (3,4,5) (PIP3)], which subsequently promotes the phosphorylation of AKT through PDK1.

Phosphatidylinositol 3-kinase is a highly conserved family of lipid kinases, which catalyzes the phosphorylation in the D3 position of the myoinositol ring of specific phosphoinositides (Vanhaesebroeck and Waterfield, 1999). A large number of intracellular functions have been attributed to class I PI3K, which comprises four different proteins in vertebrates (Foster et al., 2003). This class of PI3K can use Inositol (Ins), such as Ins (4) P and Ins (4,5) P2, as substrates (Vanhaesebroeck and Waterfield, 1999; Falasca and Maffucci, 2007). Ins (3,4,5) P3 produced by class I PI3K has all the characteristics of a second intracellular messenger involved in different cellular functions and associated with PKB/AKT pathway. Class III PI3K [also known as vacuolar protein sorting protein 34 (Vsp34)] is involved in vesicular traffic events (Falasca and Maffucci, 2007). This PI3K exclusively uses Ins, and it is responsible for the production of the major fraction of Ins (3) P (Vanhaesebroeck and Waterfield, 1999). Finally, type II PI3Ks are predominantly bound to the plasma membrane and use Ins preferentially to form Ins (3) P (Vanhaesebroeck et al., 2010). The subclass type II β PI3K (PI3KC2β) was found active in the subsets of tumors and cell lines such as acute myeloid leukemia (AML), GB, medulloblastoma (MB), neuroblastoma (NB), and small cell lung cancer (SCLC). The activity of PI3KC2β is positively correlated with cell survival as well as chemo- and radioresistance; however, the molecular mechanisms are not completely clear (Domin et al., 2005; Li et al., 2009; Boller et al., 2012).

Another interesting aspect of GB biology is the role of ion channels in malignancy (Sontheimer, 2008; Takayasu et al., 2020). Among K channels, our laboratory has contributed to define the role of calcium-activated potassium channels (KCa, Catacuzzeno et al., 2012). KCa can be distinguished into three types according to the conductance at the single-channel level: (i) the big conductance (100–200 pS) calcium- and voltage-activated K channels (BKCa), (ii) the small conductance (2–20 pS) calcium-activated K channels (SKCa), and (iii) the intermediate conductance (20–60 pS) calcium-activated K channel (IKCa). We first reported the functional expression of IKCa (Iuphar name KCa3.1) in human GB cancer (Fioretti et al., 2006), and this study refers to the current that flows through this channel population as the KCa3.1 current. KCa3.1 channel belongs to the 6TM superfamily and displays a typical pharmacological profile (Manfroni et al., 2020). Specifically, the KCa3.1 channel is blocked by TRAM-34 (analog of clotrimazole), and it is resistant to iberiotoxin, TEA, and apamine. This channel is mainly present in peripheral tissues, such as secretory epithelia, blood, and endothelial cells. KCa3.1 channels have been involved in the migration process of GB cells induced by CXCL12 (Sciaccaluga et al., 2010) and serum (Catacuzzeno et al., 2011), while they are not involved in proliferation (Abdullaev et al., 2010). In GB, the KCa3.1 channels are regulated by the Ras/Raf/MEK/ERK pathway (Fioretti et al., 2006). Recently, KCa3.1 channels were found to be involved in the radioresistance of GB, and its blockers display radiosensitizing properties (Stegen et al., 2015).

In expression systems, the activity of the KCa3.1 channel is modulated by Ins (3) P, produced through the kinase activity of the class II PI3K enzyme. In fact, PI3K inhibitors, such as LY294002 or Wortmannin, inhibit the activation of these channels. Only Ins (3) P is able to modulate the KCa3.1 channel activity, while the other phosphatidylinositol 3 phosphates [Ins (3,4,5) P3, Ins (3,4) P2, and Ins (3,5) P2] are inactive (Srivastava et al., 2005). The action of Ins (3) P appears to be mediated by the activity of nucleoside diphosphate kinase B (NDPK-B), which phosphorylates histidine 318 in the carboxy-terminal portion of the KCa3.1 channel and relieves copper blocks (Srivastava et al., 2006, 2008, 2009, 2016; Manfroni et al., 2020). PKCs have also shown to regulate the KCa3.1 ion channel. The activators phorbol myristate acetate (PMA) and inhibitors (bisindolylmaleimide) of the enzyme produce a modulation of the KCa3.1 current (Wulf and Schwab, 2002). In this study, we investigated the regulation of the KCa3.1 channel by PI3K inhibitors (LY294002 and Wortmannin) in human GB cells.

## Materials and Methods

### Cell Cultures

Human GB cell lines (U251 and GL15) were grown in a modified minimal Dulbecco medium [i.e., Dulbecco’s Modified Eagle Medium (DMEM)] supplemented with 10% Fetal Bovin Serum (FBS), 100 IU/ml of penicillin, and 100 μg/ml of streptomycin. The cells are kept in plastic flasks and culture plates (Falcon) and trypsinized every 3 days, then transferred to new plates, and kept at 37°C and 5% of carbon dioxide. For the electrophysiological experiments, 30,000 cells for culture plates were seeded, and the experiment was conducted 2–3 days after plates.

### Electrophysiological Recording

The patch-clamp recording was performed according to our previous study in whole-cell perforated and dialyzed patch-clamp configuration (Fioretti et al., 2006; Ragonese et al., 2019). Currents and voltages were amplified with a HEKA EPC-10 amplifier and analyzed with the PatchMaster and Origin 4.1 software. For online data collection, currents were filtered at 3kHz and sampled at 100 μs/point. Membrane capacitance measurements were made by using the transient compensation protocol of PatchMaster. During dialyzed recordings, the extracellular solution displays the following composition (mM): NaCl 140, KCl 5, CaCl_2_ 2, MgCl_2_ 2, MOPS 5, and Glucose 10 (pH 7.4 with NaOH). The intracellular solution was (mM) KCl 155, EGTA-K 1, MOPS 5, and MgCl_2_ 1 (pH 7.2 with KOH). During the whole-cell dialyzed experiment, the concentration of free Ca^2+^ in the intracellular solution was calculated using the “WEBMAX” program.^1^ For the electrophysiological experiments carried out to maintain a stable transduction system (Figure 4), the whole perforated configuration was chosen. In this case, the extracellular solution presents a composition (mM) of NaCl 106.5, KCl 5, CaCl_2_ 2, MgCl_2_ 2, MOPS 5, Glucose 20, and Na-gluconate 30 (pH 7.25 with NaOH), and the intracellular solution was (mM) K_2_SO_4_ 57.5, KCl 55, MgCl_2_ 5, MOPS 10, and Glucose 20 (pH 7.2 with KOH). Electrical access to the cytoplasm was achieved by adding amphotericin B (200 μM) to the intracellular solution (Fioretti et al., 2006). An amount of 1 mM of octanol, a Gap Junction blocker, and 3 mM TEA, a BKCa blocker, were added to all external solutions to avoid interferences of GAP junction and BKCa currents (Fioretti et al., 2006; Ragonese et al., 2019).

**FIGURE 1:**
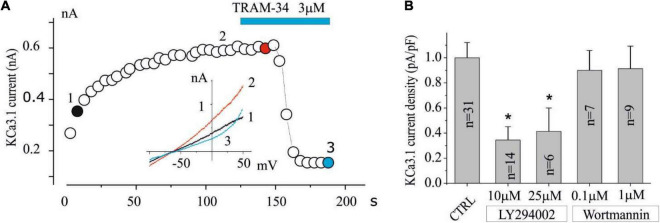
The KCa3.1 current in the U251 GB is inhibited by LY294002 but not by Wortmannin. **(A)** Exemplified time course in CTRL condition of the current at 0 mV measured by the current ramps obtained by applying linear gradients of potential from –100 to 100 mV (Vh of 0 mV) repeated every 5 s. The time when TRAM-34 is applied is also reported during recording (blue bar). Inset **(A)** Current ramps recorded in the times indicated in panel **(A)** just break of seal (1, black), after KCa3.1 current stabilization (2, red), and after 3 μM TRAM-34 block (3, green). **(B)** Bar plot of KCa3.1 current density normalized both in CTRL conditions (vehicle) and after preincubation with LY294002 (10 and 25 μM) and Wortmannin (100 nM and 1 μM) for 15 min. (*p < 0.05).

**FIGURE 2:**
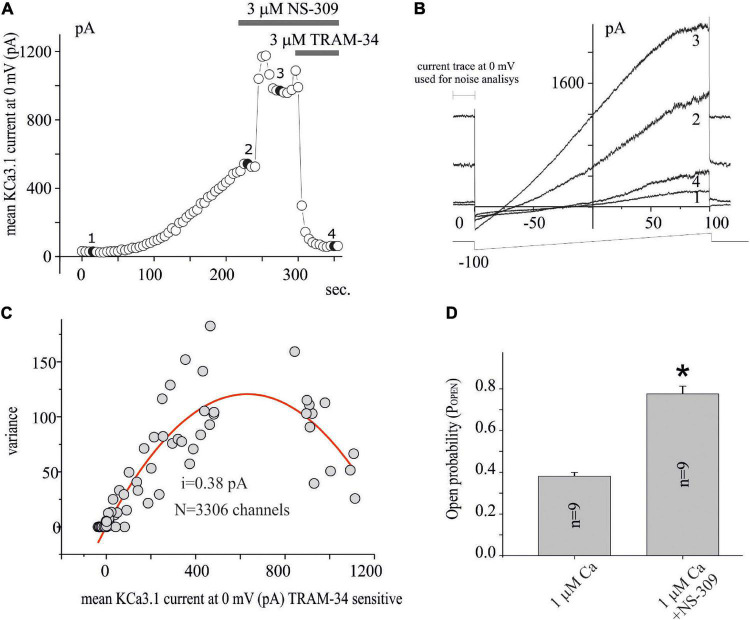
Noise analysis applied to KCa3.1 current in GB. **(A)** Time course of the current at 0 mV measured by the current ramps obtained by applying linear gradients of potential from –100 to 100 mV (Vh of 0 mV) repeated every 5 s. The times when NS-309 and NS-309 + TRAM-34 are applied, are also shown during recording (gray bars). Whole-cell configuration dialyzed with 1 μM of free calcium. **(B)** Current ramps recorded in the times indicated in panel **(A)** in the conditions CTRL (1), after KCa3.1 current stabilization (2), following 3 μM NS-309 application (3), and NS-309 + TRAM-34 co-application (4). **(C)** Plot of the variance (σ^2^) against the mean of the current (I) at 0 mV for the recording shown in panel **(A)** estimated as shown in panel **(B)**. The red line represents the parabolic fit with the function as reported in the “Materials and methods” section. **(D)** Bar plot of the P_OPEN_, estimated as reported in the “Materials and methods” section, with 1 μM Ca free in the pipette and after extracellular NS-309 application.

**FIGURE 3:**
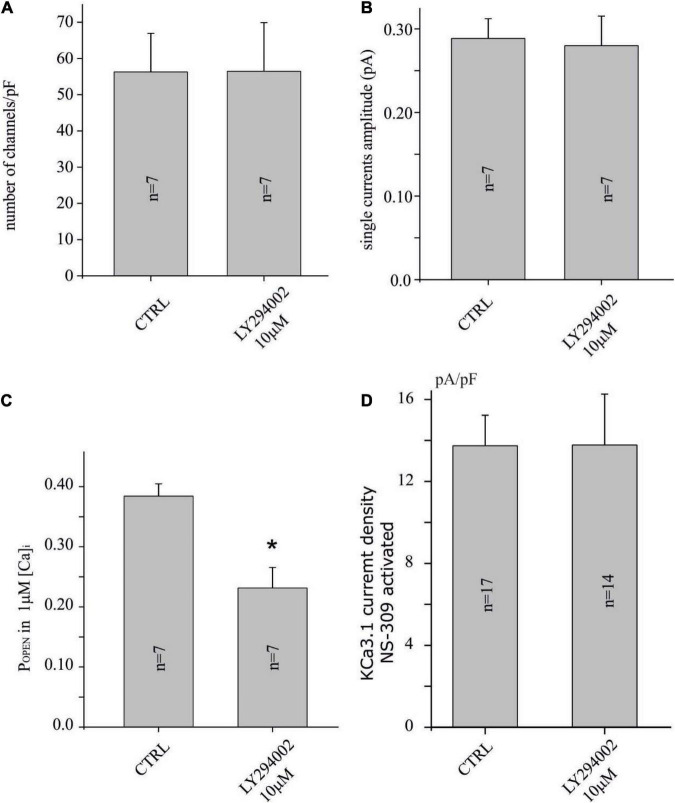
LY294002 reduces the P_OPEN_ of the KCa3.1 channel, but not the number and the amplitude of the single-channel current. **(A)** Bar plot of the number of channels (channels/pF) estimated by noise analysis in CTRL and in cells treated with LY294002 10 μM, 15 min (refer to the “Materials and methods” section and text for details). **(B)** Bar plot of the single-channel current (pA) estimated by noise analysis in CTRL and in cells treated with 10 μM LY294002, 15 min. **(C)** Bar plot of P_OPEN_ with 1 μM calcium in the pipette in CTRL condition and after treatment with 10 μM LY294002, 15 min. **(D)** Bar plot of KCa3.1 current density estimated after KCO (3 μM, NS-309) application in U251 cells in CTRL condition and treated with 10 μM LY294002, 15 min (*p < 0.05).

**FIGURE 4:**
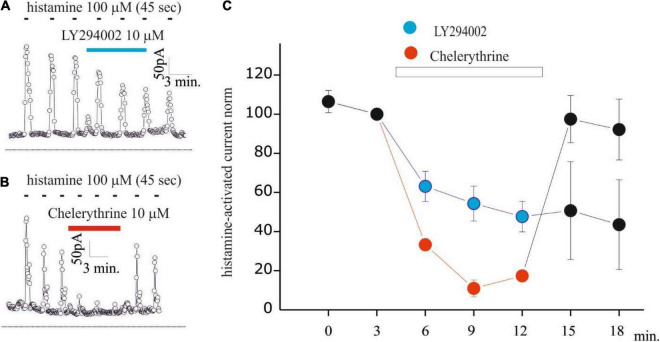
LY294002 inhibits histamine-activated KCa3.1 current in GB Gl15 cell line. **(A,B)** Time course of the current at 0 mV measured during the current ramps obtained by applying linear gradients of potential from –100 to 100 from a Vh of 0 repeated every 5 s in whole-cell perforated configuration. Histamine applications (100 μM, 45 s) repeated every 180 s and indicated by black bars, whereas blue bar indicates LY294002 10 μM (A) or chelerythrine 10 μM **(B)** application; **(C)** summary plot of the 7 experiments (in blue) performed with LY294002 (10 μM) and of the 4 experiments (in red) with chelerythrine (10 μM) on GL15 cells. All data are normalized to the histamine pre-application pulse. The histamine-activated KCa3.1 before and after (washout) the application of LY294002 and chelerythrine is shown in black dots.

All chemicals used in this study were of analytical grade. Dimethyl sulfoxide (DMSO), TEA, and octanol were purchased from Sigma Chemical Co. (St. Louis, MO, United States). NS-309 (1,6,7-Dichloro-1*H*-indole-2,3-dione 3-oxime), LY294002 [2-(4-Morpholinyl)-8-phenyl-4*H*-1-benzopyran-4-one hydrochloride], Wortmannin ((1*S*,6b*R*,9a*S*,11*R*,11b*R*) 11-(Acetyloxy)-1,6b,7,8,9a,10,11,11b-octahydro-1-(methoxymethyl)-9a,11b-dimethyl-3*H*-furo[4,3,2-*de*]indeno[4,5,-*h*]-2-*h*]-2-benzopyran-3,6,9-trione), and TRAM-34 (1-[(2-Chlorophenyl)diphenylmethyl]-1*H*-pyrazole) were purchased from Tocris Cookson Ltd. (Bristol, United Kingdom). Pharmacological agents were prepared every day in the appropriate solution at the concentrations stated and were bath applied by gravity-fed superfusion at a flow rate of 2 ml/min.

### Noise Analysis

The noise analysis, which was applied in this study for the study of the KCa3.1 channel, was conducted according to the study by Jones et al. (2007). The currents recorded for 300 ms at 0 mV were averaged to obtain the mean current sensitive TRAM-34 (I) and the variance (σ^2^). The number of channels (*N*) and the single-channel current (i) are obtained by fitting σ^2^ against the mean of the TRAM-34-sensitive current (I) according to equation 1 as follows:

(1) σ=2i-(I/2N)

The open probability (P_OPEN_) is calculated by applying equation 2 as follows:

(2) PO⁢P⁢E⁢Nx=I/xiN

### State Occupancy Analysis

The occupation of the states I, II, III, IV, and V can be interpreted by assuming that the relative occupation of the five states is based on a different stability. According to the Boltzmann equation, the relative occupation, *p*_*i*_, of a certain state *i* among a collection of *N* states, at a temperature *T* = 295 K, is given as follows:

(3) $$P_{i}=\frac{e^{-\frac{△\,E_{i}}{K\,T}}}{\sum_{j=1}^{N}e^{-\frac{△\,E_{j}}{K\,T}}},$$

where △*E*_*i*_ and △*E*_*j*_ are enthalpy changes with respect to the most stable state, and *K* is the Boltzmann constant. To be noticed that if *i* is the state V, *P*_*i*_ corresponds to P_OPEN_.

## Results

### Regulation of the KCa3.1 Current by PI3K Inhibitors

To verify the involvement of PI3K in the regulation of the KCa3.1 currents, experiments were set up by preincubating cells with LY294002 or Wortmannin. The inhibitors were applied for 15 min before the KCa3.1 current was estimated in a whole-cell dialyzed patch-clamp configuration with an intracellular free calcium concentration of 1 μM. A typical experiment is shown in Figure 1A, where the time course of the current after membrane breaking was measured at 0 mV by applying a potential ramp protocol from —100 to 100 mV (Vh = 0 mV) repeated every 5 s. As it can be noted after the breaking and the following calcium dialysis, the development of a current sensitive to TRAM-34 (KCa3.1 current) was observed. In control cells (vehicle), the KCa3.1 current density is 7.25 ± 0.9 pA/pF (*n* = 31), while current density was significantly reduced (< 0.05; 2.5 ± 0.8 pA/pF, *n* = 14) in cells treated with 10 μM LY294002. By increasing the LY294002 concentration up to 25 μM, no further decrease in current density was observed (3 ± 1.4 pA/pF, *n* = 6). In contrast, after treatment with 100 nM Wortmannin, the KCa3.1 current density does not appear to be different from the control (Figure 2B; 6.5 ± 1.2 pA/pF, *n* = 7). Furthermore, no inhibitory effects were observed when cells are incubated with 1 μM Wortmannin (Figure 2B; 6.6 ± 1.3 pA/pF, *n* = 9).

Based on the LY294002-sensitivity and Wortmannin-resistance inhibition, the involvement of PI3KC2β on the modulation of KCa3.1 current expression in U251 GB cells is suggested, and this is in agreement with expression systems (Srivastava et al., 2005). To further investigate the effects of LY294002, we applied noise analysis to estimate the impact of the treatment on the channel numbers and P_OPEN_ of KCa3.1 expressed in the U251 GB model. Figure 2A shows the time course of the current measured at 0 mV obtained as in Figure 1 during the following conditions: (i) immediately after patch breaking until the potassium current stabilization (compare point 1 and point 2 in Figures 2A,B), (ii) after the addition of Potassium Channel Openers (KCO) NS-309 (3 μM, point 3 in Figures 2A,B), and (iii) co-application with NS-309 + TRAM-34 (3 μM, point 4 in Figures 2A,B). Figure 2C shows the experimental data points reported in Figure 2A, where the average of the current TRAM-34-sensitive at 0 mV is plotted against the related σ^2^ (both parameters measured during the 300 ms before each ramp voltage application). In this figure, the fit of the data with parabolic equation (1) is reported superimposed to experimental data points. Using equation (2), the P_OPEN_ for each data point was calculated in various conditions as a ratio between the estimated currents (I_x_) and the product of the single-ion currents for the number of channels. Figure 2D shows a bar plot of P_OPEN_ estimated after the dialysis of 1 μM calcium (saturating, Fioretti et al., 2006) and following perfusion of NS-309. As observed, the noise analysis applied on 9 similar experiments indicated that 1 μM of calcium increased P_OPEN_ at about 0.40 (Figure 2D) according to the value estimated at the single-channel recording (Fioretti et al., 2006). After the application of NS-309, the P_OPEN_ increased to *ca.* 0.8 (Figure 2D).

The protocols of the noise analysis described in Figure 2 were applied in the U251 cell line in control conditions (vehicle) and after 15 min of the preincubation of 10 μM LY294002 to establish if KCa3.1 current inhibition depends on the reduction of P_OPEN_ or on the reduction of the number of channels inserted in the membrane. As it can be noted, in Figures 3A,B, the number of channels and the single-channel currents in both conditions, CTRL and LY294002 10 μM, were similar (about 60 channels/pF and 0.28 pA, respectively). In contrast, the P_OPEN_ of the KCa3.1 channel activation under 1 μM free calcium concentration is significantly reduced following the application of 10 μM of LY294002 compared to the control (Figure 3C), whereas the P_OPEN_ following NS-309 activation was not modified (P_OPEN_ in both conditions is about 0.8, Figures 2D, 3D). These data were in agreement with the involvement of PI3KC2β as a consequence of the change of the phosphorylative state of the KCa3.1 channel (Jones et al., 2007).

### LY294002 Modulates KCa3.1 Current Activated by Histamine in Human Glioblastoma Cells

Based on the inhibitory effects of LY294002 on KCa3.1 currents activated by calcium pipette dialysis, we checked whether this regulation is maintained with calcium increased by the physiological stimulus. Previously, we observed that histamine increased intracellular calcium and KCa3.1 currents in a biphasic way in GB GL15 cells, with an early peak current due to the release of intracellular calcium stores and a late plateau phase associated with calcium influx from the extracellular environment (Weiger et al., 1997; Fioretti et al., 2009). For this reason, KCa3.1 modulation was investigated in the GL15 cell line. Figures 4A,B shows the time curves of KCa3.1 currents estimated as in Figures 1, 2 during the application of histamine (100 μM, 45 s) every 180 s where a transient KCa3.1 current was observed as a consequence of washout of histaminergic stimulus (Fioretti et al., 2009). Following the application of 10 μM LY294002 (Figure 4A), a reduction in the peak current KCa3.1 was observed (Figures 4A,C). It is noteworthy that the onset of inhibition needs time (> 3 min) to reach a stable inhibition indicating the presence of a transduction process. The inhibitory effects of LY294002 were irreversible during this experiment since after washout, the KCa3.1 currents remained inhibited (Figures 4A,C). To further understand the inhibitory mechanism of LY294002 on KCa3.1 currents, we evaluated the involvement of PKC by applying 10 μM of the nonspecific inhibitor chelerythrine. Chelerythrine inhibits histamine-induced KCa3.1 currents with a major magnitude, and the effect was quickly removed after washout. Also, the onset of inhibition was much slower than that with LY294002. Altogether, these differences (magnitude, onset, and reversible) indicate that PKC is not involved in the LY294002 modulation of KCa3.1 currents in GB. Further studies are, however, necessary to understand the inhibitory effect of chelerythrine by using other inhibitors (Young et al., 1998).

## Discussion

In this study, we reported new information regarding the modulation of KCa3.1 currents in GB cancer. Specifically, we have shown that (1) KCa3.1 currents in GB are regulated by the LY294002-sensitive and Wortmannin-resistant way consistently with PI3K pathway presumably of class 2Cβ; (2) The main effect of LY294002 is the reduction of P_OPEN_ (in saturating calcium concentration), while it has no effect on the number of channels expressed on the membrane or in the amplitude of the single-channel current amplitude; (3) The regulatory effects of LY294002 are involved during the physiological activation of the KCa3.1 such as histamine stimulation; and (4) PKC activity is not involved in KCa3.1 current modulation by LY294002.

The main result of this study is the inhibitory action of LY294002 and lack of effects by Wortmannin, at the concentrations of 100 nM and 1 μM on KCa3.1 currents. This pharmacological profile suggests the involvement of PI3KC2β, which is LY294002-sensitive and Wortmannin-resistant. This is a distinctive feature when compared to the other members of the PI3K family, those of class I and III, which are LY294002- and Wortmannin-sensitive. The possible existence of class II PI3K in the U251 line was proposed to explain the resistance of vesicular traffic to Wortmannin, a process controlled by the activity of PI3Ks (Johnson et al., 2006). The involvement of class II PI3K in the regulation of KCa3.1 currents is in agreement with the results obtained by Skolnik’s group, which identifies PI3K2Cβ, which is the enzyme that regulates the KCa3.1 channel in lymphocytes (Srivastava et al., 2005). The authors also demonstrated that the action of the kinase is not direct but occurs through a phosphorylative pathway that ends with the NDPK acting on the channel (Srivastava et al., 2009). Given the indirect action of PI3K2Cβ on the KCa3.1 current, we tried to identify further phosphorylative steps that could mediate the PI3KII-dependent mechanism. For this purpose, we tested the involvement of PKCs by using a 10 μM chelerythrine inhibitor. The differences in the inhibition properties of LY294002 and chelerythrine (magnitude, onset, and reversibility) suggest that PKC is not involved in the inhibitory effect of LY2904002. However, PKCs are also involved in the regulation of the KCa3.1 current in U251 and GL15 cells. In fact, chelerythrine significantly reduces the KCa3.1 current activated by calcium dialysis (data not shown) or is physiologically stimulated by the application of histamine (Figure 4). Regarding histaminergic modulation, other mechanisms could be involved, such as the modulation of H1 receptor activity (Barajas et al., 2008).

**FIGURE 5:**
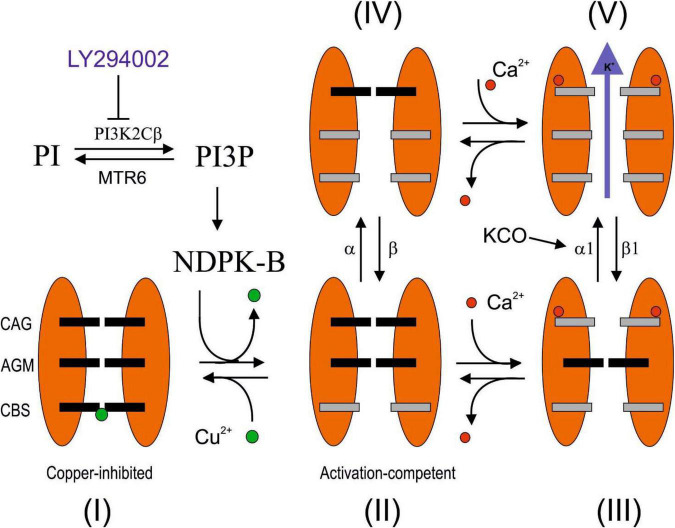
Hypothetical LY294002 inhibition mechanisms of KCa3.1 channel. The channel is described by three gates and five states (Manfroni et al., 2020). The state I is characterized by the affinity for the Cu^2+^ blocker, which is regulated by the histidine phosphorylation promoted by PI3KC2β/NDPK-B pathway and sensible to the LY294002 inhibition. In the absence of calcium, the relative occupancy of III and V is not relevant, whereas at calcium saturating concentration, the relative occupancy of II and IV turns out to be negligible. In this study, we reported three conditions at calcium saturating concentration. (1) In the absence of inhibitors, the P_OPEN_ is 0.4 since the only two possible states are III and V, and the ΔE of V with respect to III is estimated to be 1 kJ/mol, according to the Boltzmann equation. (2) In the presence of LY294002 inhibition, the P_OPEN_ is 0.2. Assuming that the difference in enthalpy between III and V is 1 kJ/mol (refer to the abovementioned details), the state I has been estimated to be the most stable with respect to III and V by 2.25 and 3.25 kJ/mol, respectively. (3) After the addition of KCO, with or without inhibitors, the P_OPEN_ is 0.8, indicating that V is the most stable state with enthalpy change by at least 3.4 kJ/mol with respect to III and I. A possible explanation that reconciles how the P_OPEN_ at the saturated calcium concentration moves from about 0.2 or 0.4 to 0.8, regardless of the inhibition by LY294002, could be justified by the stabilization of V by 4.25 kJ/mol with respect to I and 6.5 kJ/mol with respect to III (the energetics of I and III remains steady). The stabilization of V could be the consequence of the increasing of the direct constant rate α_1_ or the decreasing of the inverse constant rate β_1_. MTR6: myotubularin-related protein 6 (Srivastava et al., 2005).

The noise analysis excludes that the effect of LY294002 is to be ascribed to an internalization of the channels but rather a consequence of the reduction in P_OPEN_ from about 0.4 to 0.2 (Figure 3C) in calcium saturating concentrations (Jones et al., 2007). In agreement with the absence of internalization (number of channels in the membrane), the current estimated by the following application of potassium channel activator NS-309 is the same amplitude with a P_OPEN_ in both conditions of about 0.8 (Figures 2D, 3D). Recently, we proposed a three-gate model based on the data collected by Garneau et al. (2009), Srivastava et al. (2009), and Lee and MacKinnon (2018) in which the channel exists in five functional states. One gate was regulated by copper binding to the copper binding site (CBS); a second gate was regulated by calcium binding to the calcium activation gate (CAG), and finally, a gate was modulated by binding to KCO through the additional gating mechanism (AGM). The affinity to copper of the KCa3.1 channel depends on the histidine 358 phosphorylation by NDPK, activated by the membrane levels of InsI3(P) produced by PI3KC2b activity. Thus, InsI3 (PI3K activity) modulates copper affinity. We imagined that the channel is represented by five states (four closed states and one open state) in mutual equilibrium according to the scheme (1) adapted from Figure 1 of the study by Manfroni et al. (2020). In this new version, we specified the name of the states (open and closed) and the equilibrium process between the close state with and without copper (Figure 5). In this context, the P_OPEN_ represents the ratio between the open state (V) and the sum of the closed and open states (I+II+III+IV+V). In this scheme, NS-309 stabilized V, favoring the formation of those intermediate states toward the open state itself. In contrast, copper stabilized the closed state (I), orienting the processes toward the formation of the closed state with copper. Since InsI3 (PI3K activity) modulates copper affinity, the low P_OPEN_ observed in LY294002 at calcium saturated concentrations derives from the energetic stabilization of the closed state with copper (refer to Figure 5 for details). Further studies are needed to clarify the mechanisms of action proposed.

The subclass type II β PI3K (PI3KC2β) and the KCa3.1 channel activity were found positively correlated with cell survival as well as chemo- and radioresistance in GB cancer (Domin et al., 2005; Fioretti et al., 2006; Li et al., 2009; Boller et al., 2012; Stegen et al., 2015; Ragonese et al., 2019). For the first time, we underlined a new pathway (PI3KC2β/KCa3.1) to modulate chemo- and radioresistance during GB therapy.

## Data Availability Statement

The original contributions presented in the study are included in the article/supplementary material, further inquiries can be directed to the corresponding author.

## Author Contributions

All authors listed have made a substantial, direct, and intellectual contribution to the work, and approved it for publication.

## Conflict of Interest

RL was employed by the company Relab S.r.l. RI was employed by the company S&R Farmaceutici S.p.A Bastia Umbra. The remaining authors declare that the research was conducted in the absence of any commercial or financial relationships that could be construed as a potential conflict of interest.

## Publisher’s Note

All claims expressed in this article are solely those of the authors and do not necessarily represent those of their affiliated organizations, or those of the publisher, the editors and the reviewers. Any product that may be evaluated in this article, or claim that may be made by its manufacturer, is not guaranteed or endorsed by the publisher.

## Footnotes

1
https://somapp.ucdmc.ucdavis.edu/pharmacology/bers/maxchelator/webmaxc/webmaxcS.htm
